# Association of Anisocytosis with Markers of Immune Activation and Exhaustion in Treated HIV

**DOI:** 10.20411/pai.v2i1.199

**Published:** 2017-05-02

**Authors:** Sadeer G. Al-Kindi, David A. Zidar, Grace A. McComsey, Chris T. Longenecker

**Affiliations:** 1 Harrington Heart and Vascular Institute, University Hospitals Cleveland Medical Center, Cleveland, Ohio; 2 School of Medicine, Case Western Reserve University, Cleveland, Ohio; 3 Division of Pediatric Infectious Diseases, Rainbow Babies and Children's Hospitals, Cleveland, Ohio

**Keywords:** Red cell distribution width, anisocytosis, HIV, cardiovascular disease, inflammation

## Abstract

**Background:**

Treated HIV infection is associated with heightened inflammation which can contribute to increased risk of cardiovascular disease (CVD). We have previously shown that anisocytosis, as measured by red cell distribution width (RDW), is independently associated with prevalent CVD in people living with HIV (PLHIV). In this study, we sought to identify immune correlates of RDW in PLHIV receiving antiretroviral therapy.

**Methods:**

We performed a cross-sectional and longitudinal analysis of 147 virally-suppressed PLHIV, who had LDL < 130 mg/dL and evidence of heightened inflammation, in a randomized trial of statin therapy. A complete blood count and biomarkers of inflammation and immune activation/exhaustion were measured in peripheral blood at entry and after 24 and 48 weeks. Associations with RDW were estimated using linear regression and linear mixed models.

**Results:**

The median age (IQR) for the cohort at enrollment was 46 (40–53) years; 78% were male and 68% were African American. The median RDW for the cohort was 13.4% (12.9–14.0). Compared with the lowest RDW tertile, patients in the highest tertile were less likely to be male, and more likely to be African American, have lower hemoglobin, lower mean corpuscular volume, and higher platelet counts (all *P* < 0.05). At baseline, RDW weakly correlated with C-reactive protein (r = 0.196), d-dimer (r = 0.214), fibrinogen (r = 0.192), IL-6 (r = 0.257), CD4+DR+38+ T cells (r = 0.195), and CD4+PD1+ T cells (r = 0.227), all *P* < 0.05. Only IL-6, CD4+38+DR+ T cells, and CD4+PD1+ T_cells remained associated after adjustment for clinical factors known to affect RDW in the general population. Over 48 weeks, RDW did not change and there was no significant effect of statin (*P* = 0.45). After adjustment for clinical parameters, RDW remained positively associated with CD4+38+DR+ and CD4+PD1+ T cells across all time points (*P* = 0.05).

**Conclusion:**

In this population of treated HIV+ subjects, anisocytosis was associated with bio-markers of inflammation and T-cell activation/exhaustion over time and independent of clinical confounders. Therefore, RDW may be a useful prognostic biomarker of cardiovascular risk that partially reflects chronic inflammation and immune exhaustion in PLHIV receiving antiretroviral therapy.

## INTRODUCTION

Patients living with HIV have prevalent comorbid cardiovascular disease, higher than HIV-uninfected counterparts. We and others have shown that novel biomarkers of inflammation (eg sCD14, sCD163, CD8 T-cell activation, and IL-6) [1–5] are associated with clinical and subclinical cardiovascular disease (CVD); however, these biomarkers are generally limited to research settings and are not widely available for clinical use.

Anisocytosis is a measure of red blood cell size variability and is quantified with red cell distribution width (RDW). The parameter of RDW is routinely reported in automated blood counts and has been shown to be associated with poor prognosis in patients with heart failure and myocardial infarction [6–8]. In addition, elevated RDW is independently associated with incident CVD and mortality in the general population [9–11]. We have recently shown that RDW is independently associated with a broad spectrum of CVD in patients with HIV, including coronary artery disease, heart failure, and atrial fibrillation [12].

It has been postulated that increased RDW may reflect ineffective erythropoiesis that results from inflammation, oxidative stress, bone marrow failure, or nutritional deficiencies. Cross-sectional studies have shown that RDW correlates with markers of inflammation (eg TNF alpha, IL-6, and C-reactive protein) [6, 13, 14] in HIV-uninfected cohorts. To our knowledge, the association between RDW and immune activation in HIV has not been studied before. Given the proposed links between inflammation and cardiovascular risk in patients with HIV, we hypothesized that the relationship between RDW and CVD in HIV may be attributed in part to inflammation and/or immune activation. We performed this analysis to identify inflammatory determinants of RDW in virally-suppressed patients receiving antiretroviral therapy (ART).

## METHODS

### Study design and participants

This is a secondary analysis of data from the Stopping Atherosclerosis and Treating Unhealthy bone with RosuvastatiN in HIV (SATURN-HIV) trial (Clinicaltrials.gov NCT01218802). A total of 147 HIV-infected adults receiving ART (HIV RNA < 1000 c/ml, with a level of LDL < 130 mg/ dL, and evidence of heightened inflammation [hsCRP ≥ 2 mg/dL] or T-cell activation [CD8+CD38+HLA-DR+ ≥19%]) were randomly assigned to rosuvastatin 10 mg or matching placebo in a 1:1 design [15]. Exclusion criteria were pregnancy or lactation, immunomodulating, hormonal, or anti-inflammatory medications, any inflammatory condition besides HIV, hemoglobin level < 9 g/dL, or creatinine clearance < 50 mL/min as estimated by the Cockcroft-Gault equation. Study participants were followed for 96 weeks in the parent study; however, complete inflammation and immune activation data were only available at 0-, 24-, and 48-weeks for this analysis.

All participants provided written informed consent. The institutional review board at University Hospitals Cleveland Medical Center (Cleveland, OH) approved this study.

### RDW measurement

Venous blood was drawn at each time point for complete blood counts and was analyzed on the Sysmex XE-5000 Hematology Analyzer (Sysmex America, Inc., Lincolnshire, IL). Using electronic resistance detection enhanced by hydrodynamic focusing, the analyzer counts and sizes red blood cells. The RDW is calculated using the standard deviation of the mean corpuscular volume (MCV) divided by the mean MCV multiplied by 100.

### Comparison with NHANES

We compared our study population to a general US population by matching to a sample of HIV uninfected individuals participating in the National Health and Nutrition Examination Survey (NHANES). This is a program of studies performed by the National Center for Health Statistics (NCHS) designed to assess the health and nutritional status of the US population. The program uses a complex, multistage, probability sampling design aimed at representing the civil, non-institutionalized US population. For every cycle of NHANES (1999-current), approximately 10,000 children and adults are recruited and undergo a battery of questionnaires, physical examinations, and laboratory tests. All participants provide informed consent prior to enrollment. Complete blood counts were performed using the Beckman Coulter MAXM instruments located in the mobile examination centers.

Patients from SATURN were matched to NHANES individuals using the nearest neighbor method after sorting by age, gender, race, hemoglobin, and HIV status.

### Markers of immune activation and inflammation

Soluble biomarkers of monocyte activation (soluble CD14 and CD163), systemic inflammation (hs-CRP, IL-6, tumor necrosis factor-α receptor I [sTNFR-I]), endothelial activation (soluble vascular cell adhesion molecule-1 [sVCAM-1]), and coagulation (D-dimer and fibrinogen) were measured in batches of frozen (-80 C) plasma obtained at each time point. Soluble CD14 and soluble CD163 were measured by ELISA (R&D Systems, Minneapolis, Minnesota). Inter-assay variability ranged from 0.4% to 8.6% for soluble CD14 and from 0.7% to 18.3% for sCD163. In addition, IL-6, sTNFR-I, and sVCAM-1 were determined by quantitative sandwich ELISAs (R&D Systems and Biomedica, Vienna, Austria). Inter-assay variability ranged from 2.02% to 15.36%, from 3.66% to 5.77% and from 4.76% to 8.77%, respectively. Both Hs-CRP and fibrinogen were determined by particle-enhanced immunonephelometric assays on a BNII nephelometer (Siemens, Munich, Germany). Inter-assay variability ranged from 3.01% to 6.46% and from 3.42% to 7.59%, respectively. Levels of D-dimer were determined by immunoturbidometric assay on a STA-R Coagulation Analyzer (Diagnostica Stago, Parsippany-Troy Hills, New Jersey). Inter-assay variability ranged from 1.54% to 9.03%.

T cells and monocytes were phenotyped by flow cytometry as previously described [16], and CD4+ and CD8+ T-cell activation was defined as co-expression of CD38 and HLA-DR. Immune exhaustion was defined as expression of programmed cell death-1 (PD1) on CD4+ and CD8+ T cells. Monocyte activation was defined as the proportion of monocytes (CD14+) expressing tissue factor.

***Statistical analysis:*** Continuous variables are presented as median with interquartile range (IQR), and are compared using the Mann-Whitney U test (2 variables), Kruskal-Wallis test (> 2 variables) if non-normally distributed and Student's *t* test if normally distributed. Categorical variables are presented as numbers with percentages and compared with chi-square or Fischer exact tests. Spear-man correlations were performed between RDW and inflammatory markers. Non-normally distributed variables were then log transformed for subsequent analyses.

Using data from the baseline visit, a series of univariate linear regression models were fitted with log RDW as the independent variable. Those inflammation markers that were significantly associated (*P* < 0.05) with RDW in univariate models were then tested in separate multivariate models adjusting for several clinical confounders (age, sex, race, hemoglobin, habit of smoking, and eGFR) that were selected a priori based on existing literature.

For longitudinal analyses, a series of mixed effects models with random intercept and slope were constructed to assess (1) whether statin therapy was associated with change in RDW over time and (2) whether the clinical variables mentioned above or inflammatory markers of interest were associated with RDW over time, while accounting for the correlation between repeated measures for the same individual.

All tests are 2-sided and *P* < 0.05 was considered statistically significant. The Statistical Package for Social Sciences v21 and Stata v14.2 (StataCorp; College Station, TX) were used for this analysis.

## RESULTS

### Study population

The SATURN-HIV study enrolled 147 subjects (72 randomized to the rosuvastatin group and 75 to the placebo group). At 48 weeks, 16 patients were lost to follow-up (10 in the placebo arm and 6 in the treatment arm). All 147 participants were included in this analysis. The median age (IQR) for the cohort was 46 (40–53) years, 78% were male, and 68% were African American. The median (IQR) body mass index (BMI) was 26.7 (23.5–30.2) kg/m^2^ and the creatinine level was 0.94 (0.8–1.1) mg/dl. Of the participants 63% were current smokers and an additional 16% had been smokers in the past. The median (IQR) time since HIV diagnosis was 11 (6–17) years, duration of ART was 5 (3–10) years, and the current CD4+ T-cell count was 613 (425–853) cells/μl. In addition, 78% of patients had HIV-1 RNA < 50 copies/ml (range 20–600 copies/ml). All patients were receiving ART in accordance with trial inclusion criteria: 50% were administered protease inhibitors, 5% zidovudine, and 1% stavudine. No participants were receiving iron supplements or erythropoietin therapy.

### RDW by patient characteristic

Median RDW for the cohort was 13.4% (12.9–14.0). Compared with the lowest RDW tertile, patients in the highest tertile were less likely to be male (67% vs 94%), and more likely to be African American (91% vs 48%), have lower hemoglobin (13 vs 15 g/dL), lower MCV (89 vs 94 fL), and higher platelet counts (255 vs 213 x10^3^/μL), *P* < 0.05 for all comparisons. There was no difference in age, current or nadir CD4+ cell count, HIV or ART duration, or any of the traditional cardiovascular risk factors (*P* > 0.05 for all comparisons), Table 1.

**Table 1.** Characteristics of the SATURN-HIV study population by tertile of baseline red cell distribution width.

	Tertile 1 (< 13.1%)	Tertile 2 (13.1-13.9%)	Tertile 3 (> 13.9%)	*P*-value
Age	46 [40-53]	45 [39-54]	46 [42-52]	0.838
Male	47 (94%)	32 (74%)	36 (67%)	**0.003**
African American	24 (48%)	27 (63%)	49 (91%)	**< 0.001**
HIV parameters				
Current CD4+ count (cells/mm3)	571 [442-793]	653 [456-911]	594 [361-834]	0.333
Nadir CD4+ (cells/mm3)	188 [121-322]	180 [94-262]	158 [50-291]	0.500
HIV duration (years)	14 [7-19]	8 [6-17]	12 [6-16]	0.298
ART duration (years)	6 [3-10]	7 [3-10]	5 [3-10]	0.847
Undetectable viral load (<48 c/ml)	34 (69%)	37 (86%)	40 (74%)	0.611
Protease inhibitor	29 (58%)	17(40%)	26 (48%)	0.204
Cardiovascular risk factors				
LDL	95 [77-111]	98 [75-119]	97 [72-113]	0.863
HDL	46 [37-56]	43 [35-57]	48 [38-59]	0.265
BMI	25 [23-28]	27 [23-35]	28 [24-30]	0.102
Metabolic syndrome	8 (16%)	14 (33%)	10 (19%)	0.119
Current smoker	26 (52%)	28 (65%)	39 (72%)	0.097
Family history of MI	14 (28%)	12 (28%)	20 (37%)	0.519
10-year Framingham risk score (%)	4 [1-8]	3 [1-8]	3 [1-7]	0.467
Blood indices				
Hemoglobin (g/dL)	15 [14-16]	15 [13-15]	13 [12-15]	**0.002**
Mean corpuscular volume	94 [91-98]	91 [90-95]	89 [84-94]	**< 0.001**
Hematocrit	43 [40-45]	43 [40-45]	41 [38-44]	**< 0.001**
Mean corpuscular hemoglobin concentration	34 [34-35]	34 [33-35]	33 [32-34]	**< 0.001**
Platelet count	213 [185-262]	232 [191-271]	255 [204-315]	**0.01**
Alcohol use (current or previous)	34 (68%)	24 (56%)	35 (65%)	0.457
eGFR	93 [73-116]	104 [91-119]	104 [87-118]	**0.037**

Abbreviations: HIV = Human Immunodeficiency Virus; LDL = Low density lipoprotein; HDL = High density lipoprotein; BMI = Body mass index; MI = Myocardial Infarction; eGFR = estimated glomerular filtration rate. *P* values typed in bold denote statistical significance.

Compared with HIV-uninfected individuals from NHANES who were matched by age, gender, race, and hemoglobin concentration, HIV-infected patients had higher RDW (median 13.4% vs 12.9%, *P* < 0.001), despite these similar factors, Table 2.

**Table 2.** Comparison between HIV-infected and matched HIV-uninfected patients.

	HIV- (NHANES) (n=147)	HIV+ (SATURN) (n=147)	*P*-value
Age	44.4 ± 11.3	45.4 ± 9.9	0.42
Male	115(78%)	115(78%)	1.00
Race			1.00
White	43 (29%)	43 (29%)	
African American	100 (68%)	100 (68%)	
Hispanic	2 (1%)	2 (1%)	
Other	2 (1%)	2 (1%)	
Hemoglobin (g/dL)	14.1 ± 1.5	14.1 ± 1.5	0.99
RDW (%)	12.9 [12.4-13.4]	13.4 [12.9-14.0]	**< 0.001**

Abbreviations: HIV = Human Immunodeficiency Virus; RDW = Red Cell Distribution Width

### Association between RDW and biomarkers

**Figure 1.**
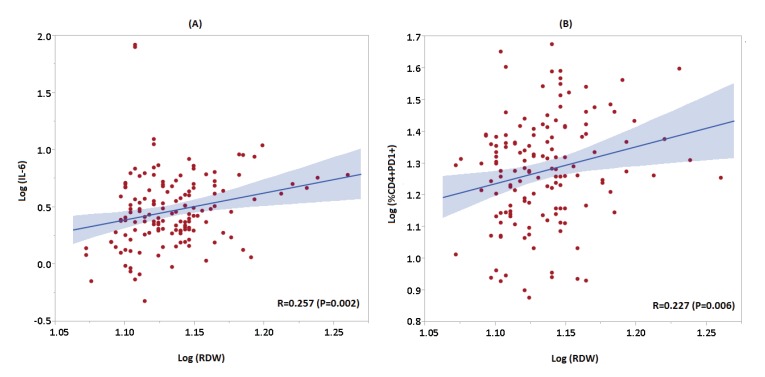
Scatter plots demonstrating the relationship between RDW and (A) log IL-6 and (B) log CD4+PD1+ T cells. Regression line with 95% CI displayed in red.

In spearman correlation analyses, RDW correlated with CRP (r = 0.196), d-dimer (r = 0.214), fibrinogen (r = 0.192), IL-6 (r = 0.257), CD4+DR+38+ (r = 0.195), and CD4+PD1+ (r = 0.227), *P* < 0.05 for all comparisons (Figure 1). Table 3 shows the correlation between biomarkers of inflammation and immune activation/exhaustion with hemoglobin and RDW. After adjustment for clinical confounders, only IL-6 (adjusted β coefficient 0.237, *P* = 0.004), percentage of CD4+38+DR+ T cells (adjusted β coefficient 0.188, *P* = 0.019), and percentage of CD4+PD1+ T cells (adjusted β coefficient 0.224, *P* = 0.008) remained associated with RDW, Table 4. The association between RDW and percentages of CD4+38+DR+ T cells and CD4+PD1+ T cells remained significant after further adjusting for total CD4+ count (adjusted β coefficient for percentage of CD4+38+DR+ 0.209, *P* = 0.02; percentage of CD4+PD1+ 0.243, *P* = 0.009).

**Table 3.** Spearman correlations between RDW and hemoglobin with baseline markers of inflammation and immune activation.

	RDW		Hemoglobin	
Baseline Marker	Rho	*P*-value	Rho	*P*-value
C-Reactive Protein	.196	**0.017**	-0.126	0.127
D-Dimer	.214	**0.009**	-0.294	**<0.001**
Fibrinogen	.192	**0.02**	-0.259	**0.002**
ICAM	0.075	0.369	0.08	0.104
IL-6	.257	**0.002**	-0.148	0.073
TNF alpha receptor I	0.011	0.9	0.135	0.104
TNF alpha receptor II	0.001	0.986	-0.044	0.598
VCAM	-0.133	0.109	0.208	**0.011**
sCD14	-0.053	0.520	-0.135	0.103
SCD163	0.099	0.231	0.07	0.399
CD14+TF+ monocytes	0.095	0.261	0.054	0.523
CD4+DR+38+ T cells	.195	**0.02**	-0.056	0.503
CD4+PD1+T cells	.227	**0.006**	-0.010	0.909
CD8+DR+38+ T cells	0.06	0.475	0.076	0.366
CD8+PD1+T cells	0.037	0.657	-0.012	0.89

RDW = Red Cell Distribution Width. *P* values typed in bold denote statistical significance.

**Table 4.** Unadjusted and adjusted linear regression models of the relationship between biomarkers of inflammation and immune activation/exhaustion and log RDW.

	Unadjusted		Adjusted* (without Hb)		Adjusted* (with Hb)	
	Beta	*P*-value	Beta	*P*-value	Beta	*P*-value
C-Reactive Protein (Log)	0.185	**0.025**	0.160	**0.045**	0.150	0.059
D-Dimer (Log)	0.191	**0.020**	0.099	0.23	0.077	0.354
Fibrinogen (Log)	0.179	**0.030**	0.088	0.30	0.074	0.385
IL-6 (Log)	0.236	**0.004**	0.255	**0.002**	0.237	**0.004**
%CD4+DR+38+ (Log)	0.203	**0.015**	0.185	**0.023**	0.188	**0.019**
%CD4+PD1+ (Log)	0.223	**0.007**	0.227	**0.008**	0.224	**0.008**
sCD14	-0.004	0.963	0.015	0.85	-0.008	0.924
SCD163	0.077	0.353	0.111	0.167	0.118	0.139
%CD14+TF+	0.122	0.149	0.112	0.166	0.126	0.117

* Adjusted for age, sex, race, smoking, and eGFR (with or without hemoglobin). *P* values typed in bold denote statistical significance.

### Longitudinal analyses

RDW did not change between baseline and week 48 (*P* = NS). Although subjects assigned to statin treatment tended to have lower RDW at baseline and across time (*P* = 0.07), there was no statin effect on RDW over 48 weeks (treatment by weeks interaction *P* = 0.456). Over time, higher RDW was associated with African American race (*P* < 0.001) and lower hemoglobin levels (*P* < 0.001). After adjustment for clinical confounders, both higher percentages of CD4+PD1+ cells (*P* = 0.050) and higher percentages of CD4+38+DR+ cells (*P* = 0.014) remained associated with RDW across all time points.

## DISCUSSION

To our knowledge, this is the first study to evaluate the association between RDW and inflammatory markers in subjects with chronic HIV infection. We show that in the absence of severe anemia, RDW is modestly associated with markers of inflammation and T-cell activation/exhaustion independently of other patient characteristics and markers.

We have previously demonstrated that RDW is independently associated with CDV (namely coronary artery disease, heart failure, and atrial fibrillation) in a large cohort of people with HIV [12]. The current findings suggest that while RDW is associated with inflammation and immune activation/exhaustion, this association is of modest strength and is not associated with one specific inflammatory pathway. Thus, the association of RDW with CVD in these patients may not fully be explained by chronic inflammation and immune activation. It is also possible that the inflamma-tory markers studied here did not adequately reflect the heightened inflammatory state caused by HIV, implicated in the pathogenesis of CVD.

It appears that RDW may serve as a composite marker of cardiovascular risk that integrates many inter-related risk factors. For example, RDW may reflect chronic inflammation but may also be related to direct bone-marrow involvement by HIV and impaired iron metabolism leading to ineffective erythropoiesis. In fact, there is some evidence suggesting that HIV infection is associated with perturbation of erythropoiesis, which in part may be related to the inflammatory milieu in HIV [17]. Interestingly, there was no association between RDW and traditional cardiovascular risk factors or the Framingham risk score in this cohort, suggesting that RDW may be able to identify patients at high cardiovascular risk independently of these traditional risk factors.

Studies have shown that in HIV-uninfected cohorts RDW correlates with erythropoietin, serum iron, ferritin, IL-6, IL-1 beta, but not TNF alpha [6]. Our observed association between RDW and both activation (38+DR+) and exhaustion (PD1+) markers on CD4+ T cells may be limited to HIV-infected cohorts, and may reflect CD4+ T cell dysfunction that persists despite viral suppression. One the other hand, the relationship between IL-6 and RDW may be causative rather than just an association, because IL-6 is a powerful inducer of hepcidin [18] which decreases ferroportin resulting in reduced iron absorption and elevation in RDW.

Our study raises the importance of studying dysfunctional erythropoiesis as a risk factor not only for AIDS but also non-AIDS comorbidity in patients with HIV. Impairment in iron mobilization has been shown to predict HIV progression [19] but it is unknown if this is associated with AIDS and non-AIDS associated co-morbidity in patients living with HIV.

Importantly, in our study, there were no significant changes in RDW over 48 weeks with or without statin therapy, suggesting that RDW can be a stable measure over time, reflecting chronic, rather than acute processes and may serve as an ‘inflammatory fingerprint' for these patients. The relative stability of RDW has been shown in other cohorts such as patients with leukemia [20] and heart failure [21]. This is in contrast to inflammatory cytokines that have a high degree of temporal variance. In addition, measuring RDW is an automated process with high test, retest reliability that can be compared across populations. Although rosuvastatin use was associated with declines in some markers of inflammation and immune activation [22] and surrogate markers of cardiovascular risk [15], there was no effect of rosuvastatin on RDW in this analysis.

These findings, in addition to the previously described association with clinical CDV, suggest that RDW may reflect a variety of pathological mechanisms ranging from inflammation and viral replication to clinical comorbidities. Thus, it may prove to be a simple, inexpensive measure of cardiovascular and non-cardiovascular morbidity in patients with HIV. It is possible that RDW may reflect immune failure, viral load set-point, and an overall trajectory of disease outcome. It is also possible that RDW may even predict overall survival in contemporary patients with HIV receiving ART, similarly to uninfected patients, but that requires further investigation.

*Limitations:* As with all secondary observational analyses of clinical trial data, our study is limited by the inability to account for unknown and unmeasured confounding factors, including iron deficiency, and erythropoietin levels. Additionally, this study is based on a subset of HIV patients who were eligible for the SATURN-HIV trial (ie mostly male, mostly African-American, virally-suppressed with ART, and with heightened inflammation); thus, the results may not be generalizable to all HIV-infected patients. Additionally, because of the cross-sectional study design, we were unable to explore causality with regards to the inflammatory pathways associated with RDW observed in this cohort.

## CONCLUSION

In this population of treated HIV-positive patients, anisocytosis is associated with biomarkers of inflammation and T-cell activation/exhaustion over time and is independent of clinical confounders. In addition, RDW may be a useful prognostic biomarker of cardiovascular risk that partially reflects chronic inflammation and immune exhaustion in PLHIV receiving ART.

## CLINICAL TRIAL REGISTRATION

NCT01218802 (www.clinicaltrials.gov)
